# Transcriptome Profiling Reveals Differential Gene Expression during the Process of Microtuber Formation in *Pinellia ternata*

**DOI:** 10.3390/ijms241411604

**Published:** 2023-07-18

**Authors:** Chen Bo, Chuandong Su, Jingtong Teng, Wei Sheng, Tao Xue, Yanfang Zhu, Jianping Xue

**Affiliations:** Anhui Provincial Engineering Laboratory for Efficient Utilization of Featured Resource Plants, College of Life Sciences, Huaibei Normal University, Huaibei 235000, China; boc2625@163.com (C.B.); scd18555969851@163.com (C.S.); ttt7510@163.com (J.T.); biosw2006@126.com (W.S.); xuetao_26@163.com (T.X.)

**Keywords:** *Pinellia ternata*, microtuber, RNA-seq, differentially expressed genes (DEGs), expression pattern

## Abstract

Using petiole material as explants and directly inducing the formation of microtubers without going through the callus stage is an essential way to rapidly expand scarce medical plants such as *Pinellia ternata*. However, the early molecular mechanism underlying the formation of the microtuber is largely elusive. Here, we conducted cytology and dynamic transcriptome analyses of inchoate microtubers in *Pinellia* explants and identified 1092 differentially expressed genes after their cultivation in vitro for 0, 5, and 15 days. Compared with 0 day, the number and size of the microtuber cells were larger at 5 and 15 days of culture. Detailed categorization revealed that the differentially expressed genes were mainly related to responses to stimulus, biological regulation, organelles, membranes, transcription factor activity, and protein binding. Further analysis revealed that the microtuber at different incubation days exhibited quite a difference in both hormone signaling pathway transduction and the regulation pattern of transcription factors. Therefore, this study contributes to a better understanding of the early molecular regulation during the formation of the microtuber and provides new insights for the study of the rapid expansion of *P. ternata* and other medical plants.

## 1. Introduction

*Pinellia ternata* (Thunb.) Breit is a valuable medical plant that belongs to the Araceae family. It is native to the eastern part of Asia and now mainly distributed in China [1,2]. The tuber of *P. ternata* is the central medicinal part, which has been frequently used in traditional Chinese medicine for thousands of years [3,4,5]. Modern pharmacological research has demonstrated that *P. ternata* tubers have anti-depressant, wound-healing, anti-coughing, and anti-vomiting functions [6,7]. In addition, alkaloid compounds, including guanosine, inosine and ephedrine, have been recognized as the main active ingredients and are believed to exert anticancer effects [8].

In recent years, with climatic and environmental changes and indiscriminate logging all intensifying, the natural sources of *P. ternata* have gradually decreased, and artificial cultivation has become mainstream. The natural seed-setting rate of *P. ternata* is very limited as it is mostly cultivated using bulbs and explants for asexual reproduction [9]. However, the former is less efficient at reproduction, and annual production is far too inadequate to meet the market demand [10]. With the advancement of plant tissue culture technology, direct induction of microtuber formation using petiole material as explants without going through the callus stage has become an important way to ensure the rapid expansion of *P. ternata* [11].

Microtubers can be produced in vitro from explants as a single node cutting or from petioles in tuber-inducing medium in small stationary-container bioreactors and fermenters, and different concentrations of plant growth regulators have different effects on the formation of microtubers in *P. ternata* [12,13]. It has been found that in vitro, *P. ternata* cell cultures produce similar amounts of alkaloid compounds to field-grown tubers [14]. Effective microtuber induction could be achieved by subculturing the morphologically expanded lower portion of suspension-cultured petioles into the solid MS medium without the addition of additional plant growth regulators. And an additional 100 μM salicylic acid (SA) increased the concentration of total alkaloid compounds, guanosine, inosine, and ephedrine by 2.5-, 2.1-, 2.8-, and 3.1-fold, respectively, suggesting that the induction of in vitro microtubers in such cultures is a promising approach for large-scale alkaloid production [15]. After making microtubers induced in the test tube into artificial seeds for cultivation, the tuber yield was twice that of cultivated seedlings, and the content of the active ingredient alkaloids had increased by 1.6-fold [16]. Hence, microtuber technology has significant advantages in tuber seed production, germplasm conservation, breeding programs, and research.

With advanced sequencing technology, a large amount of data resources and research methods have been applied to study *P. ternata*. Lu et al. (2013) [17] used EST library sequencing to identify the expression of candidate genes related to tuber development and high temperature response. Another study identified a number of potential genes associated with delayed sprout-tumble formation and tuber growth by full-length transcriptome sequencing of shade-induced *P. ternata* [18]. However, the early molecular mechanisms of *P. ternata* microtuber formation as well as the hormones and the transcription factors that are involved in the formation process are largely unknown. In this study, we performed dynamic transcriptome analysis of *P. ternata* microtuber cultured for different time intervals in order to analyze differentially expressed genes (DEGs) and to mine and screen candidate genes associated with microtuber formation. Our primary aim was to gain an overall understanding of the genes that regulate microtuber formation, which could benefit the rapid expansion and varietal improvement of *P. ternata*.

## 2. Results

### 2.1. Phenotypic Comparison of P. ternata Microtubers with Different Cultivation Times

**Figure 1:**
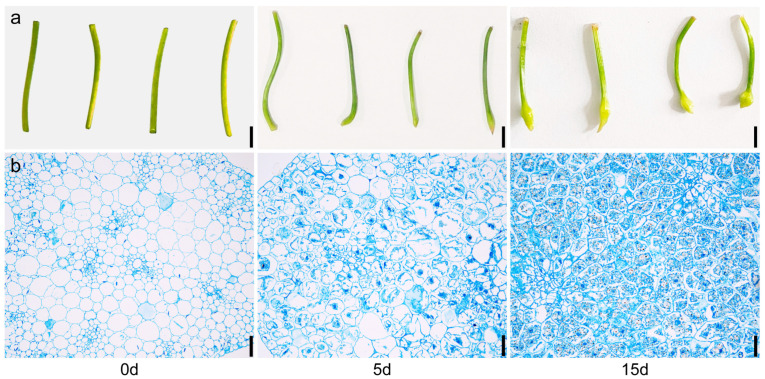
Anatomical and histological structure of *P. ternata* during microtuber formation. (**a**) Phenotype of *P. ternata* microtuber cultures at 0 d, 5 d, and 15 d. Scale bar = 5 mm. (**b**) Microscopic observations of expansion site. Scale bar = 200 μm.

Early formation of the microtuber occurs in *P. ternata* explants, with gradual expansion occurring at one end of the petiole as time progresses. As shown in Figure 1a, obvious signs of expansion were observed after 5 d of culture in Murashige and Skoog (MS) medium, and when cultured for 15 d, the average diameter of the expansion site reached 3 mm (Figure 1a). To further analyze the differences in microtubers, fresh samples (microtubers cultured for 0, 5, and 15 d) were stained with Safranin O-Fast Green, and then sectioning was performed. The changes in microtubers at different cultivation times were observed by light microscopy. Compared with 0 d, microtuber cells cultured for 5 d showed dense protoplasm. The number and size of cells increased when cultured for 15 d, and black starch granules were observed inside the cells (Figure 1b). These results suggest that the microtubers in *P. ternata* explants undergo progressive expansion and cellular changes over time. The increase in cell number, size, and the presence of starch granules indicate the development and maturation of the microtubers.

### 2.2. Transcriptome Sequencing of P. ternata Microtubers with Different Cultivation Times

**Figure 2:**
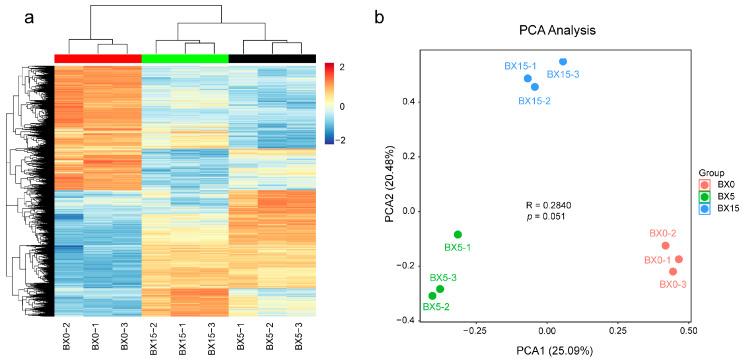
Result of RNA-seq analysis of microtubers at three different cultivation times. (**a**) Cluster analysis. The color indicates the relative levels of genes from low (blue) to high (red). (**b**) PCA analysis plot.

To further elucidate the early molecular mechanisms of microtuber formation in *P. ternata*, transcriptome analysis was performed on microtubers cultured for 0 d, 5 d, and 15 d. There were nine samples in total, BX0 (culture for 0 d), BX5 (culture for 5 d), and BX15 (culture for 15 d), with three replicates for each group. A total of 996,835 transcripts was extracted from the cDNA libraries, which were further filtered and assembled into 363,624 unigene clusters with an impressive N50 value of 610 bp (Table S1). After removing low-quality reads, the total reads and total bases produced by each library ranged from 41.37 million to 58.22 million and 59.17 million to 87.39 million. The contents of Q30 and GC were 93.48–94.88% and 52.46–53.82%, respectively, indicating the high quality of the transcriptome sequencing data (Table S2). The expression level of each gene was normalized by the transcripts-per-million-reads (TPM) method. A clustering heatmap (Figure 2a) and principal component analysis (PCA) analysis plot (Figure 2b) revealed that the gene expression data of the samples were highly reproducible between the three biological replicates of each sample, and the trends of transcriptome expression levels were similar in microtubers cultured for 5 d and 15 d, while the trends were the opposite for the 0 d samples.

### 2.3. Analysis of DEGs in BX5 vs. BX0, BX15 vs. BX0, and BX15 vs. BX5 Comparisons

**Figure 3:**
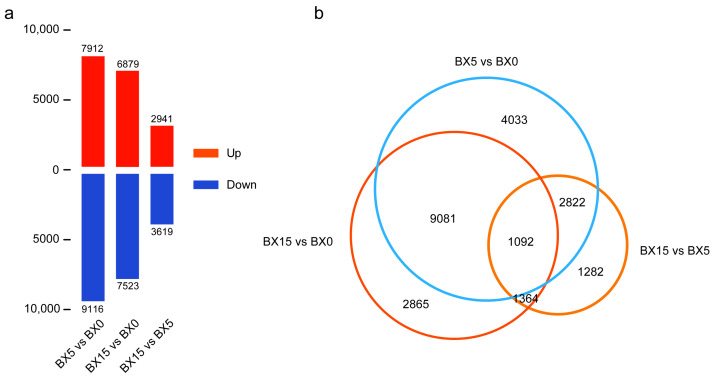
Numbers of differentially expressed genes (DEGs) in the three pairwise comparisons (**a**) and overlap between DEGs (**b**).

Each sample was compared with the other two to identify the DEGs (*q*-value ≤ 0.05 and |FoldChange| ≥ 2), and 37,990 DEGs were obtained from these three comparisons (BX5 vs. BX0, BX15 vs. BX0, and BX15 vs. BX5). From these pairwise comparisons, we identified 17,028 (7912 up- and 9116 down-regulated) DEGs in BX5 vs. BX0, 14,402 (6879 up- and 7523 down-regulated) DEGs in BX15 vs. BX0, and 6560 (2941 up- and 3619 down-regulated) DEGs in BX15 vs. BX5 (Figure 3a). Compared with the 0 d samples, the 5 d samples had the highest number of DEGs, followed by the 15 d samples, indicating that the expression of a larger number of genes are changed at the very early stage of microtuber formation than in the phase of microtuber bulking.

The Venn statistics of the comparative analysis between the groups identified 1092 DEGs that were common to microtuber formation (Figure 3b). This further indicates that DEGs associated with microtuber formation are abundantly expressed throughout the culture period and that these genes are significantly up- or down-regulated in expression during the microtuber formation phase, suggesting that the mechanisms underlying their early formation may be complex and variable. The specific expression levels and details of these DEGs are shown in Table S3.

### 2.4. Functional Classification of DEGs

A gene ontology (GO) enrichment analysis was performed in order to determine the function of the identified DEGs. The 17,028 DEGs were enriched in 3627 GO terms in the biological process categories (Table S4). Eleven GO terms included more than 1000 DEGs. Biological process (GO:0008150) and cellular process (GO:0009987), with 2514 and 2019 DEGs, respectively, were the two most dominant terms in the biological process category.

**Figure 4:**
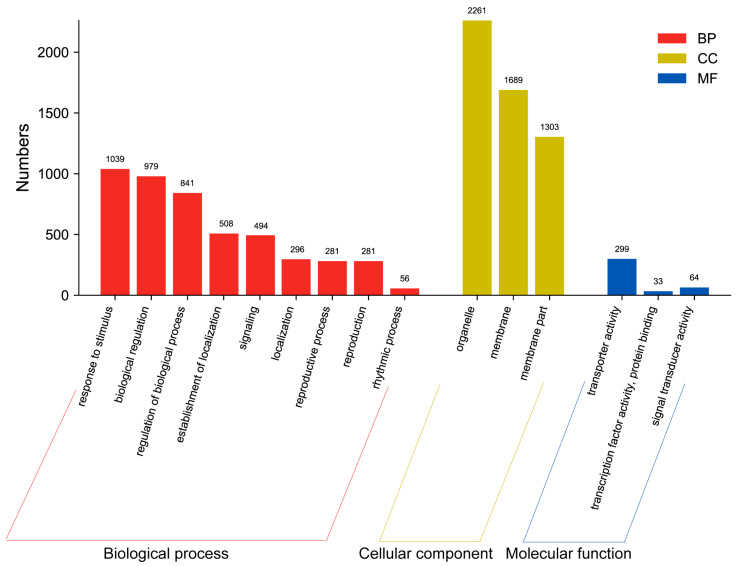
Cross-comparison of enriched GO terms among DEGs in response to microtuber formation at different cultivation times.

Among the DEGs identified among the pairwise comparisons, 15 common GO terms with a *q*-value of <0.05 were identified in the biological process category (Figure 4). As depicted in Figure 4, the significantly enriched biological processes (BPs) encompassed response to stimulus (GO:0050896), biological regulation (GO:0065007), and regulation of biological processes (GO:0050789), while the most significantly enriched cellular components (CCs) belonged to the organelle (GO:0043226), membrane (GO:0016020), and membrane parts (GO:0044425). In addition, transporter activity (GO:0005215), transcription factor activity, protein binding (GO:0000988), and signal transducer activity (GO:0004871) were significantly enriched within the molecular function (MF) categories.

**Figure 5:**
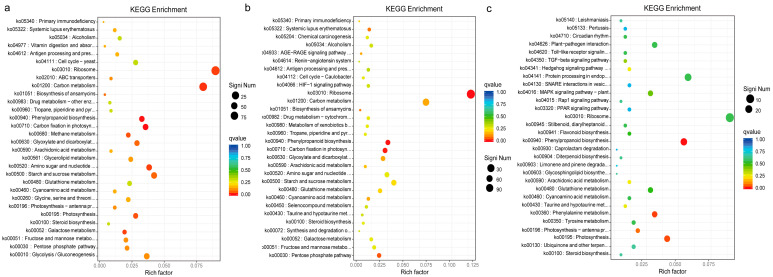
KEGG enrichment map of DEGs in BX5 vs. BX0 (**a**), BX15 vs. BX0 (**b**), and BX15 vs. BX5 (**c**) comparisons.

To further investigate the biological functions of these DEGs, pathway-based analysis was conducted using Kyoto Encyclopedia of Genes and Genomes (KEGG) pathway enrichment analysis. Cell growth and death, transport and catabolism, signal transduction, replication and repair, and translation were the topmost enriched pathways in the microtuber formation of *P. ternata* (Figure 5).

### 2.5. Analysis of DEGs Encoding Transcription Factors (TFs)

**Figure 6:**
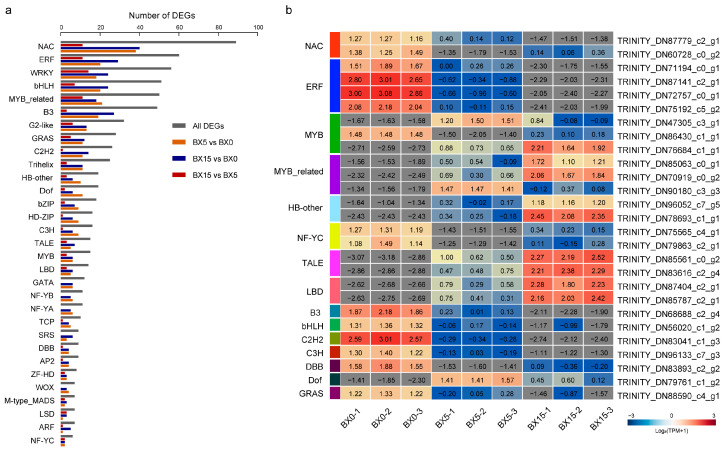
Transcription factors (TFs) encoded by DEGs detected in BX5 vs. BX0, BX15 vs. BX0, and BX15 vs. BX5 comparisons. (**a**) Classification and statistics of the TF families. (**b**) Heatmaps of the TPM+1 values of the 27 TFs.

Next, the DEGs encoding TFs were analyzed. A total of 761 DEGs encoding TFs were identified in *P. ternata* tissues that responded to microtuber formation, and these TFs belonged to 31 TF families. Most of the identified DEGs encoded members of the NAC, ERF, WRKY, bHLH, and MYB-related TF families (Figure 6a), and 22 TF families included more than 100 differentially expressed TFs. The NAC family, with 89 DEGs, was the largest TF family responding to microtuber formation in *P. ternata*, including 38, 40, and 11 DEGs in the three comparisons, respectively. A total of 60 DEGs belonging to the ERF family were identified, including 20, 29, and 11 DEGs in the three comparisons, respectively. Furthermore, a total of 56 WRKY TFs, 51 bHLH TFs, and 50 MYB-related TFs were found.

We analyzed the TFs common to the three comparison groups, and a total of 27 genes were identified. Compared with BX0, all of the common differentially expressed NACs and ERFs were down-regulated in BX5 and BX15; conversely, MYB-related and HB-other were typically up-regulated (Figure 6b). Transcription factor genes have different expression patterns in the *P. ternata* microtuber at different days of culture, suggesting that their formation processes are controlled by a combination of factors.

### 2.6. Analysis of DEGs Associated with Plant Hormones

**Figure 7:**
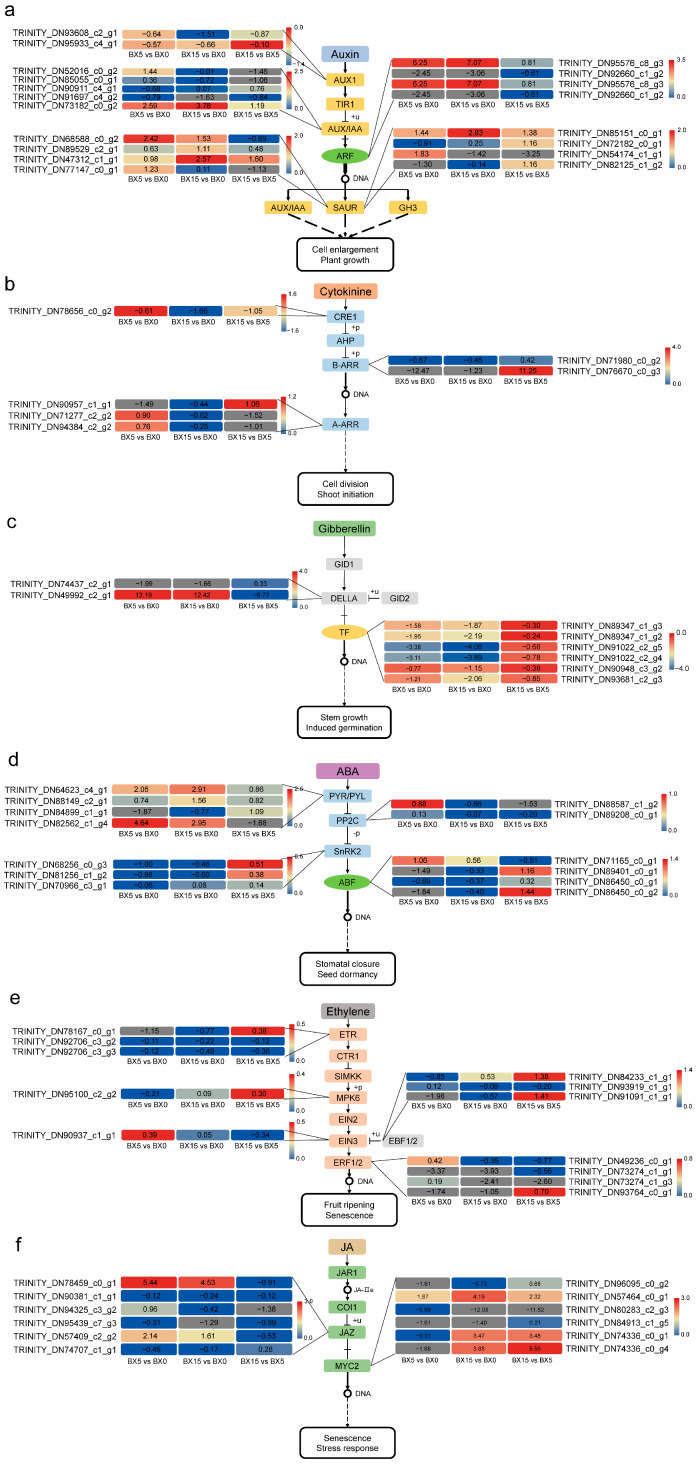
Hormone signal transduction pathways of culture-stage-specific DEGs in BX5 vs. BX0, BX15 vs. BX0, and BX15 vs. BX5 comparisons. (**a**) Auxin signal transduction pathway. (**b**) Cytokinin signal transduction pathway. (**c**) Gibberellin signal transduction pathway. (**d**) Abscisic acid signal transduction pathway. (**e**) Ethylene signal transduction pathway. (**f**) Jasmonic acid signal transduction pathway. The colored bars indicate the numbers of reads for these three comparisons (Log_2_ (TPM + 1)).

To identify crucial genes involved in important pathways, DEGs enriched in the auxin (IAA), cytokinine (CK), gibberellin (GA), abscisic acid (ABA), ethylene (ET), and jasmonic acid (JA) signaling pathways were further identified from the KEGG analysis, and about 70 DEGs involved in hormone biosynthesis and signaling transduction pathways were identified. In the IAA signaling pathway, a total of five DEGs were associated with the AUX/IAA signaling pathway, of which two were up-regulated and three were down-regulated (Figure 7a). This suggests that the transmission of growth hormone signals during microtuber formation may be multi-pathway. For DEGs associated with the CK signaling pathway, most genes were down-regulated; however, for DEGs associated with the GA signaling pathway, most were up-regulated (Figure 7b,c). These results indicate that GA and CK play distinct roles in microtuber formation. In the ABA signaling pathway, DEGs associated with the PYR/PYL, SnRK2, and ABF signaling pathways were mostly up-regulated, while two DEGs associated with PP2C were significantly down-regulated (Figure 7d). This implies that ABA has a crucial role in microtuber formation. Similarly, we also found that DEGs associated with the ET and JA signaling pathways were mostly down-regulated, suggesting they may play similar roles in microtuber formation (Figure 7e,f).

### 2.7. Validation of RNA-Seq Analysis by Quantitative Real-Time PCR (qRT-PCR)

**Figure 8:**
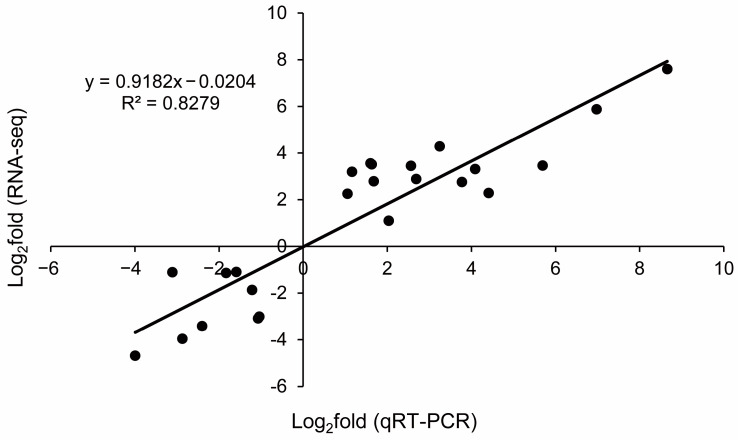
Quantitative real-time PCR validations of DEGs characterized by RNA-seq.

To validate the reliability of the gene expression data obtained by the RNA-seq analysis in the microtubers of *P. ternata*, eight DEGs that play different roles in the formation of the microtuber were selected from the three different culture-day samples for qRT-PCR analysis (Table S5). The ratio of expression levels found between the samples using qRT-PCR was compared with the ratio of expression as measured by RNA-Seq. A significant correlation (*r*^2^ = 0.8279, *n* = 24, Figure 8) was observed between the RNA-Seq and qRT-PCR data, which confirmed the authenticity of the DEGs in this study. Thus, these comparisons of data from qRT-PCR and RNA-seq analyses of microtubers fully validated the findings from our transcriptome study.

## 3. Discussion

As a valuable medicinal plant, the tuber of *P. ternata* serves as a crucial medicinal herb resource [19]. Recently, due to climate change and increased indiscriminate logging, wild *P. ternata* resources have declined, making artificial cultivation the primary method for obtaining *P. ternata* herbs. An effective approach for the rapid propagation of *P. ternata* involves using the petiole as an explant, inducing callus tissue through dedifferentiation, and then differentiating and culturing to form microtubers as propagules [20,21]. However, the early molecular regulatory mechanisms of microtuber formation remain unclear, limiting genetic improvements. 

### 3.1. Transcriptomic Analysis Yields 1092 DEGs Associated with Microtuber Formation

The identification of genes expressed during microtuber formation at the transcriptome level is an essential approach to understanding the regulatory circuits that control the microtuber formation process. This study provided cDNA sequence data from three microtuber formation stages. A total of 1092 overlapping genes that were differentially expressed in *P. ternata* explants from the three different comparisons over 15 days were focused on, and the early molecular mechanisms of microtuber formation were summarized. GO enrichment analysis revealed various biological processes, cellular components, and molecular activities relevant to *P. ternata* microtuber formation. It is reported that low auxin and cytokinin concentrations stimulate *P. ternata* microtuber polar growth, utilizing the petiole as an explant [12]. These include the response to stimulus, biological regulation, organelle function, membrane processes, as well as transcription factor activity and protein binding. Our findings provide insights into the molecular events underlying microtuber formation and shed light on the functional roles of specific genes in this process. KEGG pathway analysis showed that KEGG-enriched pathways mainly included cell growth and death, transport and catabolism, signal transduction, replication and repair, and translation. These results show that microtuber formation is a complex phenomenon involving many biological processes.

### 3.2. DEGs Encoding Transcription Factors

The highly complex biological process of tuberization can be influenced by both environmental and endogenous factors, such as photoperiod, plant growth regulators, and plantlet growth stage [22]. Understanding the regulatory mechanism of tuberization is essential for developing strategies to improve tuber yield and quality. As a result, it is necessary to conduct a more comprehensive molecular biological analysis of microtuber formation from a new perspective. Transcription factors regulate almost all aspects of plant growth and development and can orchestrate regulatory networks to improve resistance to abiotic and biotic stresses in plants [23]. Plant TF families such WRKY, NAC, AP2/ERF, bZIP, and MYB regulate numerous key biological processes throughout growth and development [24,25,26,27,28]. In this study, a total of 31 TF families containing 761 differentially expressed TFs were identified by paired comparison. Most of the differentially expressed TFs belonged to the NAC, ERF, WRKY, bHLH, and MYB TF families (Figure 6). The NAC TF family, with 89 DEGs, is the largest class that plays the largest role in the formation of the *P. ternata* microtuber, including 38, 40, and 11 DEGs in the three comparisons, respectively. A previous study indicated that ANAC043, ANAC066, and ANAC012 play key regulatory roles in secondary cell wall biosynthesis in *Arabidopsis* [29]. The identification of a large number of NAC TFs as DEGs suggests that NAC proteins may play a widespread role in the early formation of microtubers. ERF TFs are essential for floral growth, spikelet meristem destiny, root initiation, and seed/fruit development [30]. We found 20, 29, and 11 differentially expressed ERFs in each of the three comparisons during the early formation of the microtuber. Notably, AtERF38, an AP2/ERF family TF in *Arabidopsis*, was found to be involved in the regulation of several genes related to secondary wall thickening [31]. AtWRKY44 (also known as TTG2), bZIP29, and AtMYB59 were all shown to be involved in plant cell development and root growth [32,33,34]. In this study, we identified 56, 51, and 50 WRKY, bZIP, and MYB TFs involved in microtuber formation. The findings contribute to a better understanding of the early formation of microtubers and the roles of specific TF families in this process.

### 3.3. Effect of Plant Hormones on Microtuber Formation

IAA, CK, GA, ABA, ET, and JA are small-molecule plant hormones that affect many phases of plant development [35]. In this study, we observed a significant increase in cell number and size with the expansion of *P. ternata* microtubers (Figure 1), likely due to a combination of hormones. IAA is a major regulator of tuber formation and increases during early tuberization to drive cell proliferation and storage tuber formation [36]. Our transcriptome analysis revealed a total of five DEGs associated with the AUX/IAA signaling pathway, of which two were up-regulated and three were down-regulated. In *Arabidopsis*, *IAA5* and *IAA19* were rapidly and highly induced during callus induction, and they both belong to the AUX/IAA signaling pathway [37], suggesting that expression of similar genes may be highly induced during microtuber formation in *P. ternata*. The ratio of auxin to cytokinin has been suggested to be critical in determining cell fate in in vitro systems in typical plant tissue cultures [38]. Our analysis showed six DEGs involved in the CK signaling pathway, suggesting that CK may play a coordinating role for microtuber formation. GA can regulate cell elongation, which is essential for biological yield [39]. We found six transcription factor DEGs in the GA signaling pathway in this work, demonstrating that GA is essential to microtuber formation. PYR/PYL, a family of ABA receptor proteins, mediates plant immunity, seed germination, and seedling establishment [40], and our study has shown that four genes are involved in encoding PYR/PYL-type proteins, suggesting that ABA also plays a regulatory role in microtuber formation. ETH is essential for plant growth and development, including seed germination, leaf senescence, fruit ripening, and responses to environmental stresses [41]. We found genes encoding the relevant proteins on many branches of the ETH pathway, showing that the ETH pathway is crucial for microtuber formation. The JAZ protein and MYC2 are key components in the crosstalk between JA signaling and other plant hormone signaling pathways that regulates plant growth and stress responses [42]. Our investigation identified six DEGs involved in JAC and MUC2 protein coding, indicating that JA also contributes to the formation of microtubers. Although this study did not demonstrate the existence of multiple hormone signaling pathways under these experimental conditions, based on previous research and our transcriptome analysis, it can be inferred that *P. ternata* has numerous hormonal pathways associated with microtuber formation.

Taken together, our study used transcriptomic analysis to identify genes expressed during the microtuber formation of *P. ternata*, identifying 1092 DEGs and 31 transcription factor families involved in the process. Further analysis revealed DEGs associated with various hormone signaling pathways, highlighting the importance of plant hormones in the early formation of microtubers and providing insights into their complex regulatory mechanisms.

## 4. Materials and Methods

### 4.1. Plant Materials and Growth

Potted *P. ternata* plants were cultivated in a greenhouse with a 16 h/8 h light/dark photoperiod at 25 °C. The mature bulbil of healthy plants was then cut and rinsed under running water, disinfected on the surface of an ultra-clean bench with 70% alcohol for 30 s, disinfected with 0.1% mercury solution for 8–10 min, rinsed 6–7 times with sterile water, and then suspension-cultured in MS medium containing 0.2 mg/L 2,4-dichlorophenoxyacetic acid (2,4-D) and 1 mg/L kinetin (KT) until complete plants were formed. When the plants grew to a height of 4–5 cm, they were cut off at about 1.5 cm of the petiole and placed in MS medium containing 0.5 mg/L 6-benzylaminopurine (6-BA), 0.5 mg/L indolacetic acid (IAA), 5% sucrose to induce the formation of the microtuber.

### 4.2. Microscopic Structure Analysis of P. ternata Microtubers

Histological analysis was performed on microtubers of *P. ternata* sampled from three different cultivation times. Following an 8-hour fixation in FAA fixative (composed of formalin, acetic acid, and 70% alcohol in a ratio of 1:1:18), the sections of samples were rehydrated in BioDewax and put into the 0.1% safranin O staining solution for 3–8 s. Afterward, the sections were stained with 0.1% Fast Green staining solution for 6–20 s after washing away the excess dye with 1% acetic acid [43]. Imaging was performed under a Motic PA53 FS6 microscope (Motic, Hong Kong SAR, China).

### 4.3. cDNA Library Construction and Sequencing

For total RNA extraction, samples from the swollen part of the petiole were collected and frozen in liquid nitrogen at 0, 5, and 15 d after inducing microtuber formation (0.5 cm length of petiole taken close to the side of the MS medium at 0 d); samples from each cultivation time contained three biological replicates. The methods for total RNA extraction and RNA-seq library construction were the same as previously described [44]. Total RNA from the spike tissues was extracted using the TRIzol kit (Invitrogen, Carlsbad, CA, USA). The RNA was examined for quality and quantified using an agarose gel and the NanoDrop 2500 (Thermo Fisher Scientific, Wilmington, DE, USA). The mRNA was subjected to enrichment through the use of magnetic beads featuring Oligo (dT), with subsequent fragmentation through the application of fragmentation buffer to random fragments. The initial cDNA strand was produced via the utilization of small fragments as templates and arbitrary hexameric primers, which was further followed by the creation of the secondary cDNA strand using dNTPs, RNase H, and DNA polymerase I. The double-stranded complementary DNA (cDNA) was purified through the use of magnetic beads, followed by an end-repair process that involved the addition of a single nucleotide A (adenine) to the 3′ ends and subsequent ligation with sequencing adaptors. The appropriate fragments were selectively chosen as templates and enriched through PCR amplification. The quality and concentration of the RNA were evaluated using an Agilent 2100 Bioanalyzer (Agilent Technologies, Santa Clara, CA, USA). Equal amounts of RNA from each sample were sequenced on an Illumina HiSeq 2500 sequencer (Illumina, San Diego, CA, USA) at the Sangon Biotech company (Shanghai, China) to construct the strand-specific RNA-seq libraries. 

### 4.4. Data Processing and De Novo Assembly

The original data produced by the sequencer were defined as raw reads. Raw reads from each sample were filtered by removing reads containing more than 10% of unknown nucleotides, low-quality reads that contained more than 40% of low-quality (Q value ≤ 20) bases, and adapter sequences according to the Illumina adapter list. The remaining high-quality reads were assembled de novo using the Trinity software (version 2.14.0) [45]. 

### 4.5. Functional Annotation of Unigenes and Differential Expression Genes Analysis

The assembled unigenes were aligned with a non-redundant protein sequence (Nr), cluster of orthologous groups of proteins (COG), gene ontology (GO), Kyoto encyclopedia of genes and genomes (KEGG), and SwissProt databases, as well as highly similar proteins to annotate the unigenes IDs. Gene expression level read-count data was analyzed using DESeq2 for differential genes [46]. Genes with a false discovery rate (FDR) of <0.05 and |log2FC| > 1 were considered significant DEGs. Using Blast2go software (version 2.3.5, http://www.blast2go.de/, 16 July 2023), the significantly different unigenes were compared with the KEGG and GO databases for annotation and enrichment analysis. The results of functional annotation and pathway enrichment were combined to screen for candidate genes related to microtuber formation.

### 4.6. Validation of RNA-seq Data by qRT-PCR

Eight DEGs were selected for validation by qRT-PCR. Single-stranded cDNA was obtained using the Evo MMLV RT Premix for qRT-PCR (Accurate Biotechnology Co., Ltd., Changsha, China) according to the manufacturer’s protocol. Gene-specific primers were designed using Primer3Plus (http://www.primer3plus.com/, accessed on 6 December 2022). FastStart Essential DNA Green Master (Roche, Basel, Switzerland) was used for the PCRs. The reaction system and procedure are described in a previous paper [47]. The *Pt18SrRNA* gene was used as an internal control for normalization [48], and three technical replicates of each cDNA sample were analyzed. The primer sequences for qRT-PCR are listed in Table S4. The 2^−ΔΔCT^ method was used to calculate the relative expression level of each gene [49].

## 5. Conclusions

We analyzed RNA-seq to provide a global overview of the differences in the transcriptomes of microtubers from *P. ternata* at 0 d, 5 d, and 15 d of formation. A total of 1092 common DEGs were identified in microtubers at the three different culture times. The analysis of DEGs not only revealed that a large number of genes encoding TFs were highly induced during this process, but also identified some DEGs associated with hormones during microtuber formation. This will provide new insights for studying microtuber formation and rapid propagation in *P. ternata*.

## Data Availability

The raw data supporting the conclusions in this article are available from the GenBank GEO database under accession number PRJNA989437 (https://www.ncbi.nlm.nih.gov/sra/PRJNA989437, accessed on 2 July 2023).

## Supplementary Materials

The supporting information can be downloaded at: https://www.mdpi.com/article/10.3390/ijms241411604/s1.

## References

[1] Moon B., Kim W., Ji Y., Lee Y., Kang Y., Choi G. (2016). Molecular identification of the traditional herbal medicines, Arisaematis Rhizoma and Pinelliae Tuber, and common adulterants via universal DNA barcode sequences. Gen. Mol. Res..

[2] Zhang J.Y., Guo Q.S., Zheng D.S. (2013). Genetic diversity analysis of Pinellia ternata based on SRAP and TRAP markers. Biochem. Syst. Ecol..

[3] Mao Z.C., Peng Z.S. (2003). Progress on research of rapid propagation system of *Pinellia ternata*. China J. Chin. Mater. Med..

[4] Zeng X.Q., Peng Z.S. (2008). Growth and propagation of wild *Pinellia ternata* in cultivation. China J. Chin. Mater. Med..

[5] Iwasa M., Iwasaki T., Ono T., Miyazawa M. (2014). Chemical composition and major odor-active compounds of essential oil from PINELLIA TUBER (dried rhizome of *Pinellia ternata*) as crude drug. J. Oleo Sci..

[6] Kong X., Yang M., Abbas H.M.K., Wu J., Li M., Dong W. (2018). Antimicrobial genes from Allium sativum and *Pinellia ternata* revealed by a Bacillus subtilis expression system. Sci. Rep..

[7] Zhang Z.H., Zhao Y.Y., Cheng X.l., Dai Z., Zhou C., Bai X., Lin R.C. (2013). General toxicity of *Pinellia ternata* (Thunb.) Berit. in rat: A metabonomic method for profiling of serum metabolic changes. J. Ethnopharmacol..

[8] Duan Y.B., Lu F., Cui T.T., Zhao F.L., Teng J.T., Sheng W., Zhang A.M., Xue J.P. (2017). Effects of abiotic elicitors MeJA and SA on alkaloids accumulation and related enzymes metabolism in *Pinellia ternata* suspension cell cultures. Chin. J. Inf. TCM.

[9] Liu Y., Hui R., Deng R., Wang J., Wang M., Li Z. (2012). Abnormal male meiosis explains pollen sterility in the polyploid medicinal plant *Pinellia ternata* (Araceae). Genet. Mol. Res..

[10] Eguchi T., Tanaka H., Yoshida S., Matsuoka K. (2019). Temperature effects on the yield and quality of the medicinal plant *Pinellia ternata* Breit. Environ. Conv. Biol..

[11] Tsay H., Gau T., Chen C. (1989). Rapid clonal propagation of *Pinellia ternata* by tissue culture. Plant Cell Rep..

[12] Wang J., Wang Q., Wang J., Lu Y., Xiao X., Gong W., Liu J. (2009). Effect of different plant growth regulators on micro-tuber induction and plant regeneration of *Pinellia ternate* (Thunb) Briet. Physiol. Mol. Biol. Plants.

[13] Akita M., Ohta Y. (1998). A simple method for mass propagation of potato (*Solanum tuberosum* L.) using a bioreactor without forced aeration. Plant Cell Rep..

[14] Liu Y., Liang Z., Zhang Y. (2010). Induction and in vitro alkaloid yield of calluses and protocorm-like bodies (PLBs) from Pinellia ternata. Vitr. Cell. Dev. Biol.-Plant.

[15] Duan Y.B., Zhang H., Meng X., Huang M.M., Zhang Z.Y., Huang C.H., Zhao F.L., Xue T., Xue J.P. (2009). Accumulation of salicylic acid-elicited alkaloid compounds in in vitro cultured *Pinellia ternata* microtubers and expression profiling of genes associated with benzoic acid-derived alkaloid biosynthesis. Plant Cell Tissue Organ Cult..

[16] Yikun H., Changfu Z., Mengyuan H., Alin H., Shui H. (1997). Morphogenesis of tubercles and production of artificial seeds in *Pinellia ternata*. Acta Agron. Sin..

[17] Lu H., Xue T., Zhang A., Sheng W., Zhu Y., Chang L., Song Y., Xue J. (2013). Construction of an SSH library of *Pinellia ternata* under heat stress, and expression analysis of four transcripts. Plant Mol. Biol. Rep..

[18] Xue T., Zhang H., Zhang Y., Wei S., Chao Q., Zhu Y., Teng J., Zhang A., Sheng W., Duan Y. (2019). Full-length transcriptome analysis of shade-induced promotion of tuber production in *Pinellia ternata*. BMC Plant Biol..

[19] Gombodorj S., Yang M.H., Shang Z.C., Liu R.H., Li T.X., Yin G.P., Kong L.Y. (2017). New phenalenone derivatives from *Pinellia ternata* tubers derived *Aspergillus* sp.. Fitoterapia.

[20] Jian X. (2004). Research on Direct Formation of Microtubers from *Pinellia ternata*. Acta Agron. Sin..

[21] Li C. (2007). Formation of Microtubers from the Petiole of *Pinellia ternata* (Thunb.) Berit. in vitro and Change of Endogenous Hormones Content. J. Huazhong Agric. Univ..

[22] Li J., Zhao X., Dong Y., Li S., Yuan J., Li C., Zhang X., Li M. (2020). Transcriptome analysis reveals key pathways and hormone activities involved in early microtuber formation of *Dioscorea opposita*. BioMed Res. Int..

[23] Golldack D., Li C., Mohan H., Probst N. (2014). Tolerance to drought and salt stress in plants: Unraveling the signaling networks. Front. Plant Sci..

[24] Yu Y., Qi Y., Xu J., Dai X., Chen J., Dong C.H., Xiang F. (2021). *Arabidopsis* WRKY71 regulates ethylene-mediated leaf senescence by directly activating EIN2, ORE1 and ACS2 genes. Plant J..

[25] Hu P., Zhang K., Yang C. (2019). BpNAC012 positively regulates abiotic stress responses and secondary wall biosynthesis. Plant Physiol..

[26] Ohto M.A., Fischer R.L., Goldberg R.B., Nakamura K., Harada J.J. (2005). Control of seed mass by APETALA2. Proc. Natl. Acad. Sci. USA.

[27] Fukazawa J., Sakai T., Ishida S., Yamaguchi I., Kamiya Y., Takahashi Y. (2000). Repression of shoot growth, a bZIP transcriptional activator, regulates cell elongation by controlling the level of gibberellins. Plant Cell.

[28] Haga N., Kobayashi K., Suzuki T., Maeo K., Kubo M., Ohtani M., Mitsuda N., Demura T., Nakamura K., Jürgens G. (2011). Mutations in *MYB3R1* and *MYB3R4* cause pleiotropic developmental defects and preferential down-regulation of multiple G2/M-specific genes in *Arabidopsis*. Plant Physiol..

[29] Singh S., Koyama H., Bhati K.K., Alok A. (2021). The biotechnological importance of the plant-specific NAC transcription factor family in crop improvement. J. Plant Res..

[30] Feng K., Hou X.L., Xing G.M., Liu J.X., Duan A.Q., Xu Z.S., Li M.Y., Zhuang J., Xiong A.S. (2020). Advances in AP2/ERF super-family transcription factors in plant. Crit. Rev. Biotechnol..

[31] Lasserre E., Jobet E., Llauro C., Delseny M. (2008). AtERF38 (At2g35700), an AP2/ERF family transcription factor gene from *Arabidopsis thaliana*, is expressed in specific cell types of roots, stems and seeds that undergo suberization. Plant Physiol. Biochem..

[32] Johnson C.S. (2002). TRANSPARENT TESTA GLABRA2, a Trichome and Seed Coat Development Gene of Arabidopsis, Encodes a WRKY Transcription Factor. Plant Cell.

[33] Van Leene J., Blomme J., Kulkarni S.R., Cannoot B., De Winne N., Eeckhout D., Persiau G., Van De Slijke E., Vercruysse L., Vanden Bossche R. (2016). Functional characterization of the *Arabidopsis* transcription factor bZIP29 reveals its role in leaf and root development. J. Exp. Bot..

[34] Xie Z., Lee E., Lucas J.R., Morohashi K., Li D., Murray J.A., Sack F.D., Grotewold E. (2010). Regulation of cell proliferation in the stomatal lineage by the *Arabidopsis* MYB FOUR LIPS via direct targeting of core cell cycle genes. Plant Cell.

[35] Davies P.J. (2010). The Plant Hormones: Their Nature, Occurrence, and Functions.

[36] Roumeliotis E., Kloosterman B., Oortwijn M., Kohlen W., Bouwmeester H.J., Visser R.G., Bachem C.W. (2012). The effects of auxin and strigolactones on tuber initiation and stolon architecture in potato. J. Exp. Bot..

[37] Xu K., Liu J., Fan M., Xin W., Hu Y., Xu C. (2012). A genome-wide transcriptome profiling reveals the early molecular events during callus initiation in *Arabidopsis* multiple organs. Genomics.

[38] Skoog F., Miller C.O. (1957). Chemical regulation of growth and organ formation in plant tissues cultured in vitro. Cheminform.

[39] Wang X., Li J., Ban L., Wu Y., Wu X., Wang Y., Wen H., Chapurin V., Dzyubenko N., Li Z. (2017). Functional characterization of a gibberellin receptor and its application in alfalfa biomass improvement. Sci. Rep..

[40] Wang Z., Ren Z., Cheng C., Wang T., Ji H., Zhao Y., Deng Z., Zhi L., Lu J., Wu X. (2020). Counteraction of ABA-mediated inhibition of seed germination and seedling establishment by ABA signaling terminator in *Arabidopsis*. Mol. Plant.

[41] Kendrick M.D., Chang C. (2008). Ethylene signaling: New levels of complexity and regulation. Curr. Opin. Plant Biol..

[42] Li C., Shi L., Wang Y., Li W., Chen B., Zhu L., Fu Y. (2020). *Arabidopsis* ECAP is a new adaptor protein that connects JAZ repressors with the TPR2 co-repressor to suppress jasmonate-responsive anthocyanin accumulation. Mol. Plant.

[43] Wang L., Xie X., Xu Y., Li Z., Xu G., Cheng L., Yang J., Li L., Pu W., Cao P. (2022). Comprehensive analysis of the carboxylesterase gene reveals that *NtCXE22* regulates axillary bud growth through strigolactone metabolism in tobacco. Front. Plant Sci..

[44] Zhang X., Liu J., Huang Y., Wu H., Hu X., Cheng B., Ma Q., Zhao Y. (2022). Comparative transcriptomics Reveals the molecular mechanism of the parental lines of maize hybrid An’nong876 in response to salt stress. Int. J. Mol. Sci..

[45] Grabherr M.G., Haas B.J., Yassour M., Levin J.Z., Thompson D.A., Amit I., Adiconis X., Fan L., Raychowdhury R., Zeng Q. (2011). Full-length transcriptome assembly from RNA-Seq data without a reference genome. Nat. Biotechnol..

[46] Love M.I., Huber W., Anders S. (2014). Moderated estimation of fold change and dispersion for RNA-seq data with DESeq2. Genome Biol..

[47] Bo C., Cai R., Fang X., Wu H., Ma Z., Yuan H., Cheng B., Fan J., Ma Q. (2022). Transcription factor ZmWRKY20 interacts with ZmWRKY115 to repress expression of *ZmbZIP111* for salt tolerance in maize. Plant J..

[48] Zhang H., Zhang Z., Xiong Y., Shi J., Chen C., Pan Y., Xue T., Xue J., Duan Y. (2021). Stearic acid desaturase gene negatively regulates the thermotolerance of *Pinellia ternata* by modifying the saturated levels of fatty acids. Ind. Crops Prod..

[49] Livak K.J., Schmittgen T.D. (2001). Analysis of relative gene expression data using real-time quantitative PCR and the 2^−ΔΔCT^ method. Methods.

